# Robot Communication: Network Traffic Classification Based on Deep Neural Network

**DOI:** 10.3389/fnbot.2021.648374

**Published:** 2021-03-19

**Authors:** Mengmeng Ge, Xiangzhan Yu, Likun Liu

**Affiliations:** School of Cyberspace Science, Harbin Institute of Technology, Harbin, China

**Keywords:** traffic classification, capsule neural network, encrypted traffic, network security, deep learning

## Abstract

With the rapid popularization of robots, the risks brought by robot communication have also attracted the attention of researchers. Because current traffic classification methods based on plaintext cannot classify encrypted traffic, other methods based on statistical analysis require manual extraction of features. This paper proposes (i) a traffic classification framework based on a capsule neural network. This method has a multilayer neural network that can automatically learn the characteristics of the data stream. It uses capsule vectors instead of a single scalar input to effectively classify encrypted network traffic. (ii) For different network structures, a classification network structure combining convolution neural network and long short-term memory network is proposed. This structure has the characteristics of learning network traffic time and space characteristics. Experimental results show that the network model can classify encrypted traffic and does not require manual feature extraction. And on the basis of the previous tool, the recognition accuracy rate has increased by 8%

## Introduction

With the rapid development of technology, humanoid robots can do more things on behalf of people, such as helping people guide paths, serving coffee, and turning on lights. While humanoid robots liberate people's labor, there are also some risks of security and privacy leakage in these processes.

Robots need to interact with people or server commands when they are working (Gleeson et al., 2013; Mavridis, 2015). When robots and people interact through voice, everyone can hear the commands issued by people. When people want to hide the behavior and content of the commands, people can Use codes instead, such as a cough that means a command to turn on the light. This is the easiest way to hide the content of communication between humans and robots. When the server communicates with the robot, it is impossible for the server to cough and issue a command like a human (Su et al., 2020). He will put the control command in the network message and send it to the robot in a specific protocol format. When a stranger repeatedly observes the behavior of coughing, the robot will light up. He speculates that the coughing behavior may correspond to the command to turn on the light. Therefore, by observing the communication process between the control server and the robot in the network, and through learning and training, the communication protocol between the server and the robot can be identified, and further, the command line in the communication process between the server and the robot can be inferred as the type (Kanda et al., 2010). In this article, we have studied the protocol identification of network messages, which can identify the type and protocol of network communication traffic, which is of great significance to the discovery of malicious network attack traffic in the communication process of humanoid robots. It can also be used for web traffic detection (Tian et al., 2020) and IoT traffic detection (Shafiq et al., 2020).

The development of traffic classification technology has gone through three stages: port-based, payload-based, and flow-based statistical characteristics. Port-based classification methods infer the types of mobile services or applications by assuming that most applications always use “well-known” TCP or UDP port numbers (Li et al., 2000; Hjelmvik and John, 2009; Wang et al., 2017). However, the emergence of port masquerading, random ports, and tunneling technologies quickly lost these methods Effectiveness. The payload-based method, that is, DPI (Deep Packet Inspection) (Moore and Papagiannaki, 2005; Finsterbusch et al., 2013; Wang et al., 2019) technology cannot handle encrypted traffic because it needs to match the content of the data packet and has a high computational overhead (Madhukar and Williamson, 2006). In order to try to solve the problem of encrypted traffic identification, a flow-based method has emerged, which usually relies on statistics or time series features and uses machine learning (Zuev and Moore, 2005; Liu et al., 2007; Fan and Liu, 2017; Shafiq et al., 2018) algorithms, such as Naive Bayes, Support Vector Machine (Li et al., 2007; Yuan et al., 2010; Groleat et al., 2012; Ebrahimi et al., 2017), Decision tree, random forest (Siahaan et al., 2019), k nearest neighbor (KNN)(Este et al., 2009; Wu et al., 2015; Sun et al., 2018). In addition, some statistical models, such as Gaussian Mixture Model (Alizadeh et al., 2015; Kornycky et al., 2016; Pacheco et al., 2018) and Hidden Markov Model (Yin et al., 2012), are used to identify and classify encrypted traffic.

Although classic machine learning methods can solve many problems that cannot be solved by methods based on ports and payloads, it still has some limitations: (1) It is difficult to obtain manually extracted traffic characteristics, and these characteristics always depend on domain experts' experience. Therefore, it is impossible to automatically extract and select features, which will cause great uncertainty and confusion in classic machine learning methods when ML is applied to mobile service traffic classification. (2) Flow characteristics are easily outdated quickly and need to be constantly updated. (3) How to combine a large, easily accessible unlabeled data set with some expensive labeled data sets for traffic classification to reduce the need for labeled data is a very critical research topic. (4) For traffic classification tasks, category imbalance is not a small problem. However, current data enhancement methods cannot accurately generate samples as close to the original data distribution as possible.

Unlike most traditional machine learning algorithms, deep learning (Gu et al., 2020) can perform automatic feature extraction without manual intervention. This paper uses the algorithm based on the capsule convolutional neural network (Vinayakumar et al., 2017; Rezaei and Liu, 2019) and the self-attention LSTM neural network to identify the encrypted network traffic (Fu et al., 2016; Si et al., 2019). The results show that this method does not require manual feature extraction and has excellent classification effects.

## Related Background Content

### Capsule Neural Network

**Figure 1:**
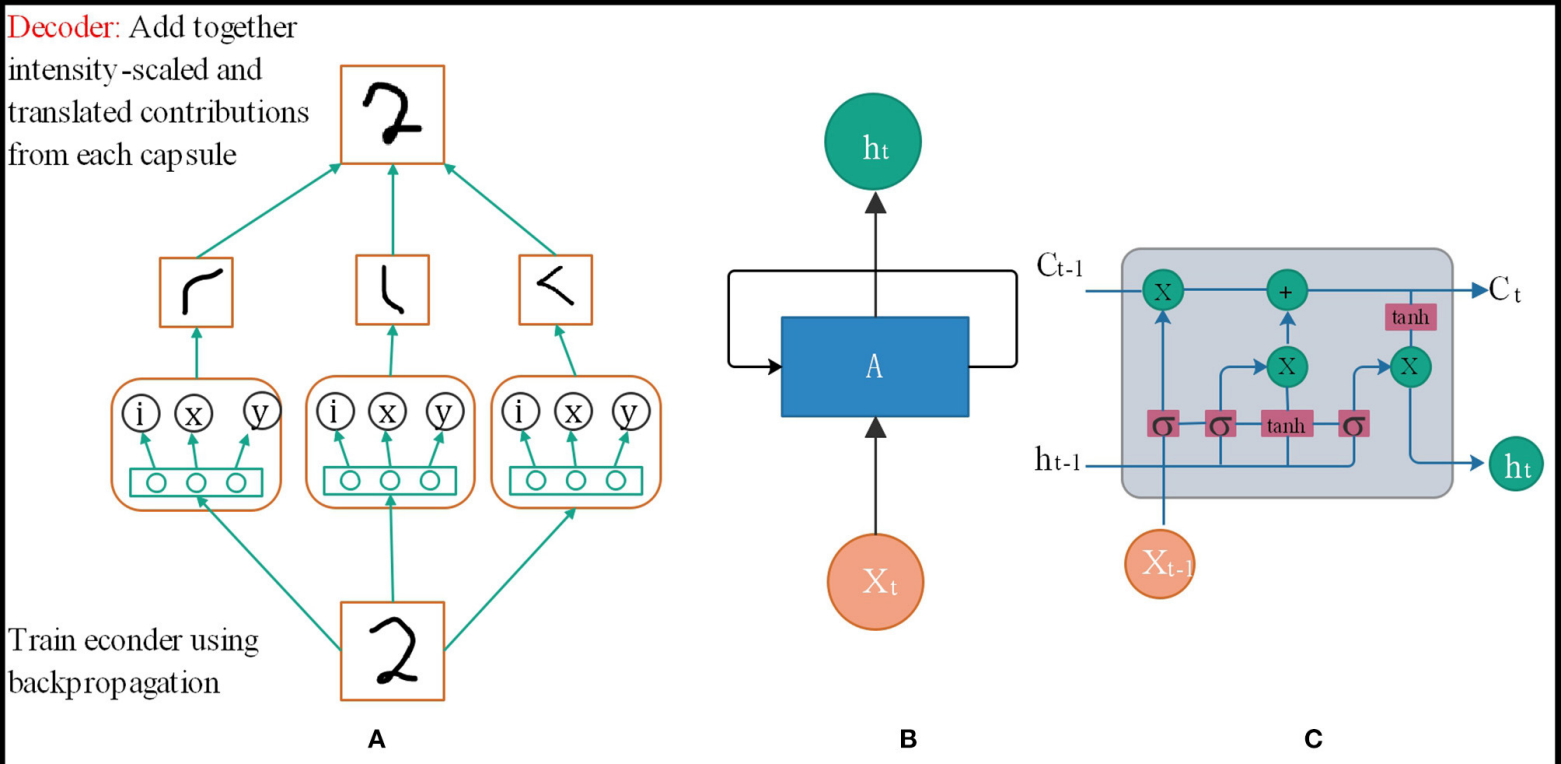
**(A)** Capsule neural network structure. **(B)** RNN neural network structure. **(C)** LSTM neural network structure.

Convolutional Neural Networks (CNN) have good image recognition performance, but they still have some shortcomings. When the photos are crowded and blurred, the classification effect will worsen, and the output of the model does not respond well to small changes in the input. The Capsule Network (CapsNet) (Xiang et al., 2018) solves some of the traditional neural convolutional network problems because the capsule network is composed of directional neuron groups, capsules instead of neurons. The traditional training feature of each neuron, learned which area in the spatial feature is not fixed, it is entirely random, but in the capsule network, each neuron group learns a fixed area in a certain area in the picture Features, such as the eyes and nose of a human face. The capsule network is also composed of multiple layers. As shown in Figure 1A, the vector capsule is at the bottom of the network (Zhu et al., 2019). The capsule network (Deng et al., 2018) also has a perceptual domain, just like the traditional CNN. However, for each vector capsule, their perceptual domain is a fixed part of the spatial feature, and they only learn the features of that region. During the training process, the parameters are modified continuously to improve the accuracy of classification and recognition. Further, some small capsules will be gathered into large capsules, called routing capsules, to learn more extensive spatial features. For example, vector capsules identify network traffic packet space features, and routing capsules identify the space of multiple network traffic packets. And then, the characteristics of the entire flow or stream are recognized.

#### Anti-fragility

The capsule network output is not the individual value output by the traditional artificial neuron but the output vector. The parameters of the dynamic combination of different vector capsules in the routing can be calculated through its unique dynamic routing mechanism, which can be trained for different perspectives.

#### Robustness

Redundant homomorphism can solve complex problems. Many small features will be trained by one Capsule. Although the parameters between capsules are independent of each other, many capsules will train similar substructures. Simultaneously, the high-level capsule can learn the relationship between the various structures of the bottom-level capsule. Even though the image to be recognized becomes blurred or shifted and other perspective changes, it can be correctly recognized through this redundant network structure.

#### Interpretability

It is possible to know what each capsule is responsible for training and which sub-structural features each capsule recognizes so that each parameter of the neural network is no longer a complete black box.

### Self-Attention Long Short-Term Memory

When analyzing the flow characteristics between network sessions, it can be found that it is more like a sentence that conforms to the special established rules, except that it is composed of bytes to form a data packet and then a session. It is very similar to the structure of natural language problems where words form sentences and sentences form paragraphs. The principle of using a time series neural network to identify which application a session belongs to is also similar to the principle of sub-classification. The essence is to use the form of an array instead of the data itself as input. A similar time-series relationship of similar data is obtained through the model to achieve classification.

As shown in Figure 1B, the traditional RNN network model cannot fully handle the timing problem because it needs to determine the parameters before it can be predicted, but in the process of training the parameters of the neural network model, the chain derivation rule to determine the gradient increment of the parameter is essential of. In this case, when the problem sequence to be dealt with is relatively long, it will be relatively large, and the upper bound of the derivative of the common activation function is, so when it is very large, there will be many less than the numerical value in the chain derivation rule. When the time sequence is very long, the parameter increments updated by the chain derivation rule will be close, causing the parameters to be unable to be updated. In other words, RNN can handle timing problems, but when the sequence of timing problems is very long, the information cannot be very effective Was saved, so researchers proposed an improved model LSTM[38]. The network structure is shown in Figure 1C.

The reason why LSTM can solve the long time sequence problem of *t* is because the cell state *C*_*i*_ is used to assist in the transfer of data to time *t*, instead of relying on the hidden layer information at time *h*_*t*−1_, and the update formula of cell state *C*_*i*_ can also be used. Knowing that it is updated through addition. The advantage of this is that when the chain rule is updated, the cell state *C*_*i*_ will not appear as the updated parameter increment in the RNN chain derivation rule will be close to 0.

The attention mechanism was first proposed in the field of visual images, and has gradually been widely used in natural language since 2014. Today, the combination of various attention mechanisms and deep learning network models has achieved good results in natural language problems. As a result, this article also considers adding a self-attention mechanism when using the time-series deep learning model LSTM to process traffic.

Self-attention (Tao et al., 2020). The above process can be abstracted as the calculation of similarity between query, key and value, which can be roughly divided into three stages:(1) query and *key*_*i*_ use the similarity function that matches the task to calculate the similarity, and get the parameter *s*_*i*_. (2) Normalize *s*_*i*_ with *softmax*() to get α_*i*_. (3) After multiplying the corresponding α_*i*_ and *value*_*i*_, and then summing, the self-attention value can be finally obtained. The calculation process is as follows:

$$\begin{array}{l} f\left(Q,K_{i}\right)=Q^{T}K_{i}\\ \alpha_{i}=softmax\left(f\left(Q,K_{i}\right)\right)=\frac{\exp\left(f\left(Q,K_{i}\right)\right)}{\sum_{i}\exp\left(f\left(Q,K_{i}\right)\right)}\\ self-attention\;\left(Q,K,V\right)=\underset{i}{\sum }\alpha_{i}V_{i}\left(Q=K=V\right) \end{array}$$

## Framework and Methods

Our framework logically consists of two parts: the “pre-processor” and the “traffic classifier.” The former has performed all tasks that allow us to model the network traffic into data, which can easily be handled by a deep learning model. The latter performs specific classification tasks. Before using a deep learning traffic classifier, it must be trained with a large amount of labeled traffic data. The traffic data we use comprises the public dataset ISCX2012 and the data generated by artificially stimulating the apps.

### Network Traffic Pre-processing

**Figure 2:**
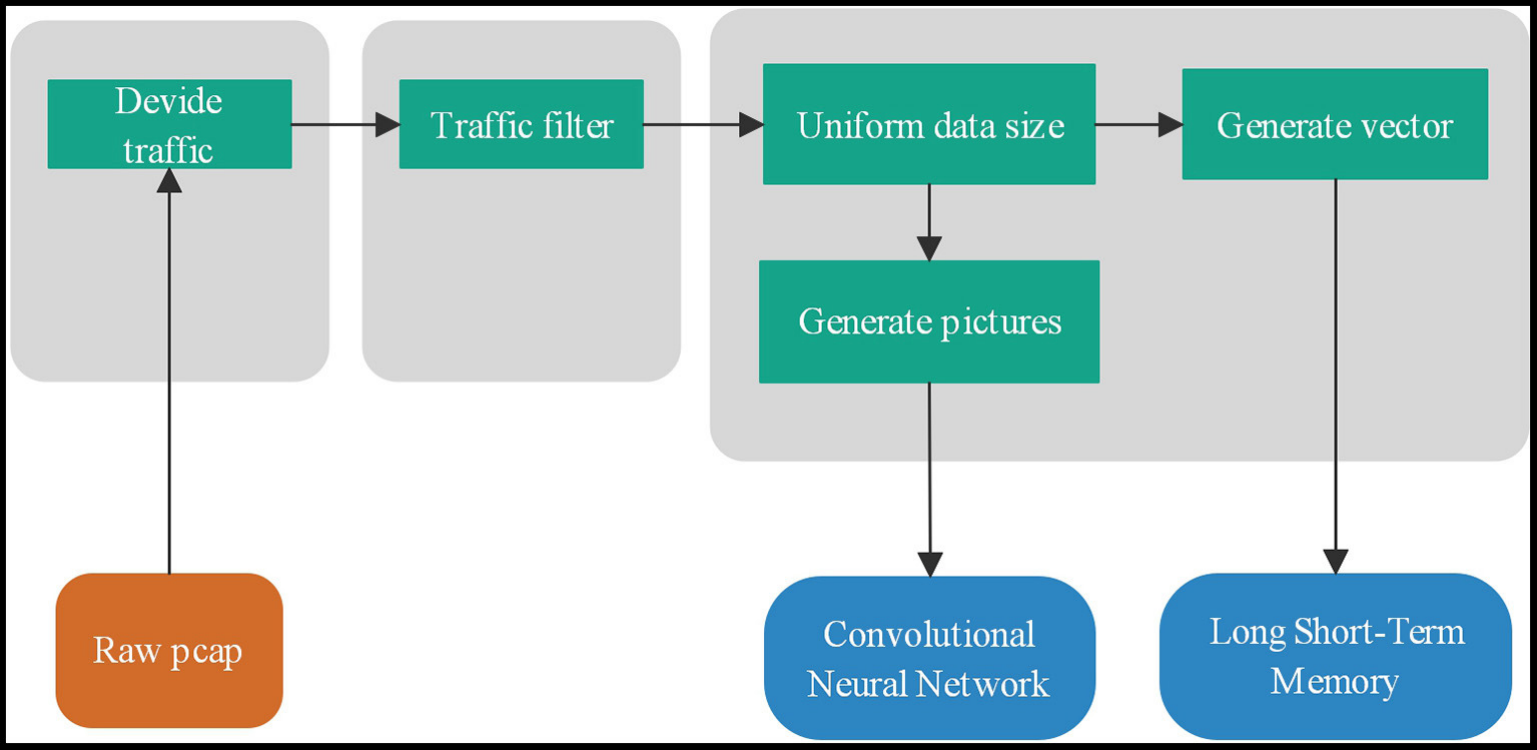
Network traffic data packet processing process: flow division, flow filtering, uniform size and classification.

There are two traffic forms: flow and session. Usually, we use five tuples to determine flow and session. Flow: a time-ordered sequence of packets exchanged between two peers during a single TCP session. f = < *p*_1_, *p*_2_, …, *p*_*N*_ >, N is the number of packets consist of flow f. packet *p*_*i*_= (*a*_*i*_, *l*_*i*_, *t*_*i*_). *a*_*i*_ is a 5-tuple that consists of source address, source port, a destination address, destination port, and the protocol type. *l*_*i*_ is the length of the packet *p*_*i*_ and *t*_*i*_ is the time of packet *p*_*i*_ arrival. The total flow length L = $\sum_{i=1}^{n}l_{i}$ and *t*_1_ ≤ *t*_2_, …, ≤ *t*_*n*_. Session and flow are similar. The difference is that the source and destination addresses can be exchanged, which is a bidirectional data flow. Network traffic can be divided into four levels, application layer, transport layer, network layer, and all layers. The input data of the traffic classifier, a combination of different traffic forms and different network layers, are eight types. The pre-processing is shown as Figure 2.

### Packers Filtering

Due to network congestion, traffic load balancing, or other unpredictable network behaviors, data packets may be lost and arrive out of order. When TCP detects these problems, it will retransmit network data packets and rearrange out-of-order data packets. Repeated transmission packets will affect the characteristics of network traffic, and network flows without data content will not use in identification. Therefore, we filter out retransmissions and packets with only ACK flags with zero payloads. The sequence of network packets has an essential impact on recognition. We will rearrange out-of-order data packets to obtain a correct network flow sequence.

### Traffic Data Processing

Traffic classification should remove data related to the hardware environment and network environment, such as IP information in the network layer and MAC addresses in all layers. Therefore, training data and test data may have different physical addresses and IP addresses. We need to remove these features that may change to ensure the feature consistency of the training data and test data.

The input length of the classifier is consistent to ensure the correct subsequent recognition. At the same time, the selected data length has a significant impact on the recognition result. Through analysis of the contribution of bytes in the stream, we found that the first 400 bytes in the stream have an impact on the traffic classification because the recognition contribution is the highest. Therefore, we choose 400 as the input of the convolutional neural network. Data streams with a length of <400 will be dropped, and data streams with a length of more than 400 bytes will only take the first 400 bytes of the data stream as classifier input.

Many previous research works have shown that CNN neural network has a good classification performance for image recognition. Here we take the first 400 bytes of a network stream, and we can get a 20^*^20 two-dimensional matrix. Each value range of the matrix is 0–255, so each value can be regarded as the gray value of a pixel. Thus, picture data containing the first 400 bytes of information in the data stream can be obtained. When the network traffic is processed and converted into pictures, we get the features of the traffic stream. This paper uses the CapsuleNet neural network, capsule represented by a vector can learn the spatial feature relationship of the flow graph well.

### Traffic Classification

#### Convolutional Neural Network

The first 400 bytes of encrypted data traffic is converted into a 20^*^20 grayscale image, which is the input of the traffic classifier. The Inception-CapsuleNet network designed in this paper is divided into nine layers. The first four layers extract the characteristics of the traffic, the middle four layers combine the characteristics, and the last layer is the category output layer, as shown in Table 1. Before the grayscale image entry the model, the mean value is zeroed first so that the model converges quickly.

**Table 1:** Capsule convolutional neural network.

**Layer**	**Name**	**Activation function**	**Input size**	**Convolution kernel**	**Step**	**Output size**
1	Condv2	ReLU	20*20*1	9*9*256	1	12*12*128
2	Batch Norm	–	12*12*128	–	–	12*12*128
3	Inception	ReLU	12*12*128	–	–	12*12*256
4	Primary Caps	Squash	12*12*256	6*6*256*8	2	4*4*8*32
5	DigitCaps	Squash	4*4*8*32	–	–	10*12
6	Full Connect	ReLu	10*12	–	–	256
7	Full Connect	ReLU	256	–	–	128
8	Full Connect	ReLU	128	–	–	64
9	Full Connect	Softmax	64	–	–	8

The first layer is a convolutional layer, which extracts local features of grayscale images. In order to learn more about the local features of the input data, the step size is 1. The second layer uses batch normalization, which can prevent the data distribution from changing greatly after passing through the previous layer, and can avoid the gradient disappearance and overfitting problems. In the third inception layer, convolution kernels of different sizes are used for feature processing.

Convolutions of different sizes can extract features from different images in different fields of view, which can increase the ability of the network to extract features. Finally, the outputs of different convolution kernels are spliced together to obtain a feature with a dimension of 256. There are 32 capsules in the PrimaryCaps layer, each of which will convolve all the inputs of the previous layer. Here the activation parameter is squash, and an out tensor is *u*_*i*_, with a shape of 4^*^4^*^8^*^32. The input data in the DigitCaps layer is the vector *u*_*i*_. The calculation process is as follows:

$$\begin{array}{l} {\hat{u}}_{j|i}=w_{ij}\cdot u_{ij}\\ b_{ij}=b_{ij}+{\hat{\text{u}}}_{j|i}\cdot v_{j}\\ c_{ij}=softmax\;\left(b_{ij}\right)=\frac{\exp\left(b_{ij}\right)}{\sum_{k}\exp\left(b_{ik}\right)}\\ s_{j}=\sum c_{ij}\cdot {\hat{\text{u}}}_{j|i}\\ v_{ij}=\frac{\|s_{j}{\|}^{2}}{1+\|s_{j}{\|}^{2}}\cdot \frac{s_{j}}{\left\|s_{j}\right\|} \end{array}$$

Where *s*_*j*_ is the final input, the final output vector is *v*_*j*_ and $b_{ij}^{\left(0\right)}=0$. After the activation function Squash, *b*_*ij*_, *c*_*ij*_ can be updated by Equations (2) and (3). The best parameter selection can be achieved by continuously repeating the above process. At the same time, in order to prevent over-fitting, the number of iterations here is selected as three, and finally, ten capsules are output, and each capsule is a 12-dimensional vector.

The following are three fully connected layer classification networks, and finally, a vector of length 64 is obtained, and the last one is a fully connected softmax activation function classifier.

#### Long Short-Term Memory Network

In order to make full use of the characteristics of the network flow, the network structure of this article first uses the self-encoding method to train the data packets into a unified array specification as the information representing the encrypted flow. Then use the time series neural network to extract the timing behavior characteristics of the data packet exchange process at both ends of the conversation, and use the characteristics to classify the encrypted network traffic of the application. Because the flow is in the process of data packet exchange, the encrypted content of the current data packet may be determined by the protocol in a previous data packet. The pure LSTM time series network cannot well capture the characteristic information generated by this behavior. This article will add from The attention mechanism allows each data packet unit to better correspond to its own related data packets during the training process, hoping to obtain more comprehensive network traffic characteristics and achieve higher recognition results.

**Figure 3:**
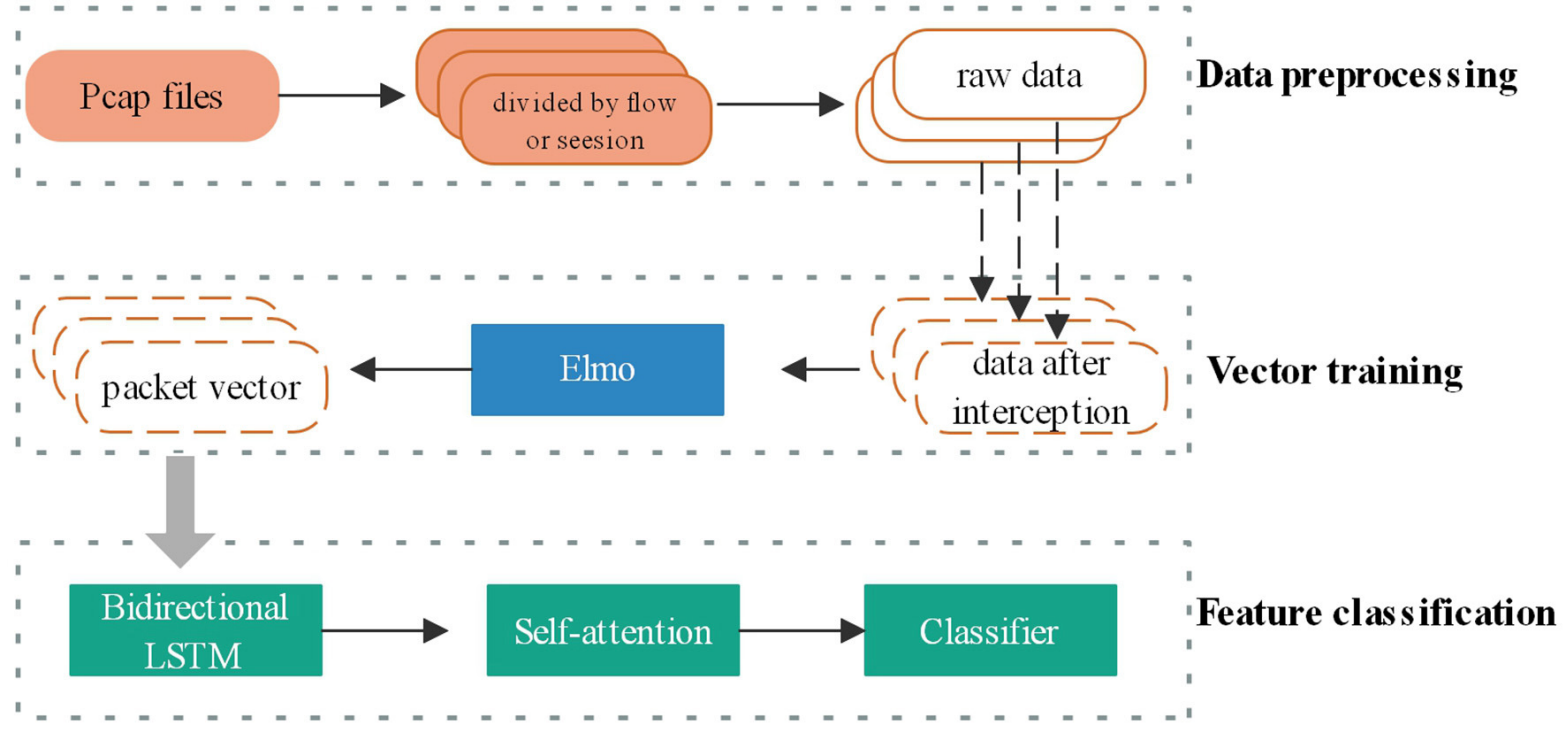
LSTM neural network data processing process.

The data processing process is shown in Figure 3. Before the model training is carried out, after the traffic is cleaned, the data packets are sorted according to the flow or session according to the packet sending time of the packet header. After the traffic enters the model, first select the number of reserved data packets, then select the reserved byte length of each data packet, and then convert multiple data packet bytes into an array vector through the method of self-encoder. Finally, it enters the model's feature extraction classifier training stage.

In the stage of training the word vector, the traditional method is to use the word form to represent the word vector. But when the number of dictionaries is too long, the word vector cannot be used in deep learning algorithms. The relevance of the word vector is very poor, so this article uses the Distributed method to train the word vector. The vector obtained by this method can be controlled to a shorter length.

When training the word vector of the data packet, it is considered that the meaning of the same byte in the encrypted traffic protocol of different applications is very different. If a traditional word vector training method, such as word2vec is used to obtain a fixed word vector, this does not conform to the characteristics of the data in the encrypted traffic recognition model. Elmo's two-layer bidirectional LSTM network pre-training structure can obtain word vectors that meet the complexity of the protocol, and the same byte corresponds to different word vectors in different protocol environments.

Regarding the hexadecimal bytes in the pcap packet, this article converts it into decimal data as the original input, and then intercepts the 96 bytes of the first six packets of each stream or session as input. Less than 96 bytes, using the method of adding 0 to fill in, the form of the array converted into an array of 6^*^96 dimensions. Its form is similar to the way of analyzing a sentence category in text classification. For a 6-word sentence, the word vector length of each word is fixed at 96. In form, it is similar to the first 6 data contained in a stream or conversation. The information contained in the data packet represents that the two ends of the conversation are communicating through a certain language, so it is reasonable to input it into the training model of natural language word vector Elmo.

In the Elmo stage, the Embedding of the first layer of LSTM and the Embedding of the second layer of LSTM are multiplied by the corresponding weights, and the final Embedding is < *t*_1_, *t*_2_, …, *t*_*n*_ > Then, where *n* represents the number of data packets, the value here is 6, that is, 6 data packets are selected, and each data packet intercepts 96 bytes as input. The size of the LSTM of the Elmo stage is 128, that is, the size of the vector *t*_1_ length, and then input Elmo's result data into the LSTM+Self-Attention model.

After entering the LSTM+Sefl-Attention stage, after the first layer of LSTM, the activation function is Relu, the word vector feature of the data packet is extracted and the size is 256 as the input of Batch Normalization to ensure that the data distribution remains unchanged while maintaining 256 The length remains unchanged and enters the next layer of Self-Attention. After this network layer, you can learn an encrypted session or stream. The internal structure of the intercepted data packets and the dependency relationship between the protocol features help The model identifies and classifies encrypted traffic. After passing through the Sefl-Attention network layer, the traffic length remains unchanged at 256, and then passes through a layer of Batch Normalization to enter the fully connected layer. The number of neurons in the first fully connected layer is 64, the activation function is Relu, and the second fully connected layer. The number of neurons is 7, the activation function is Softmax, which is used to finally output the probability that the encrypted traffic belongs to the target application.

#### CNN-LSTM Joint Network

**Figure 4:**
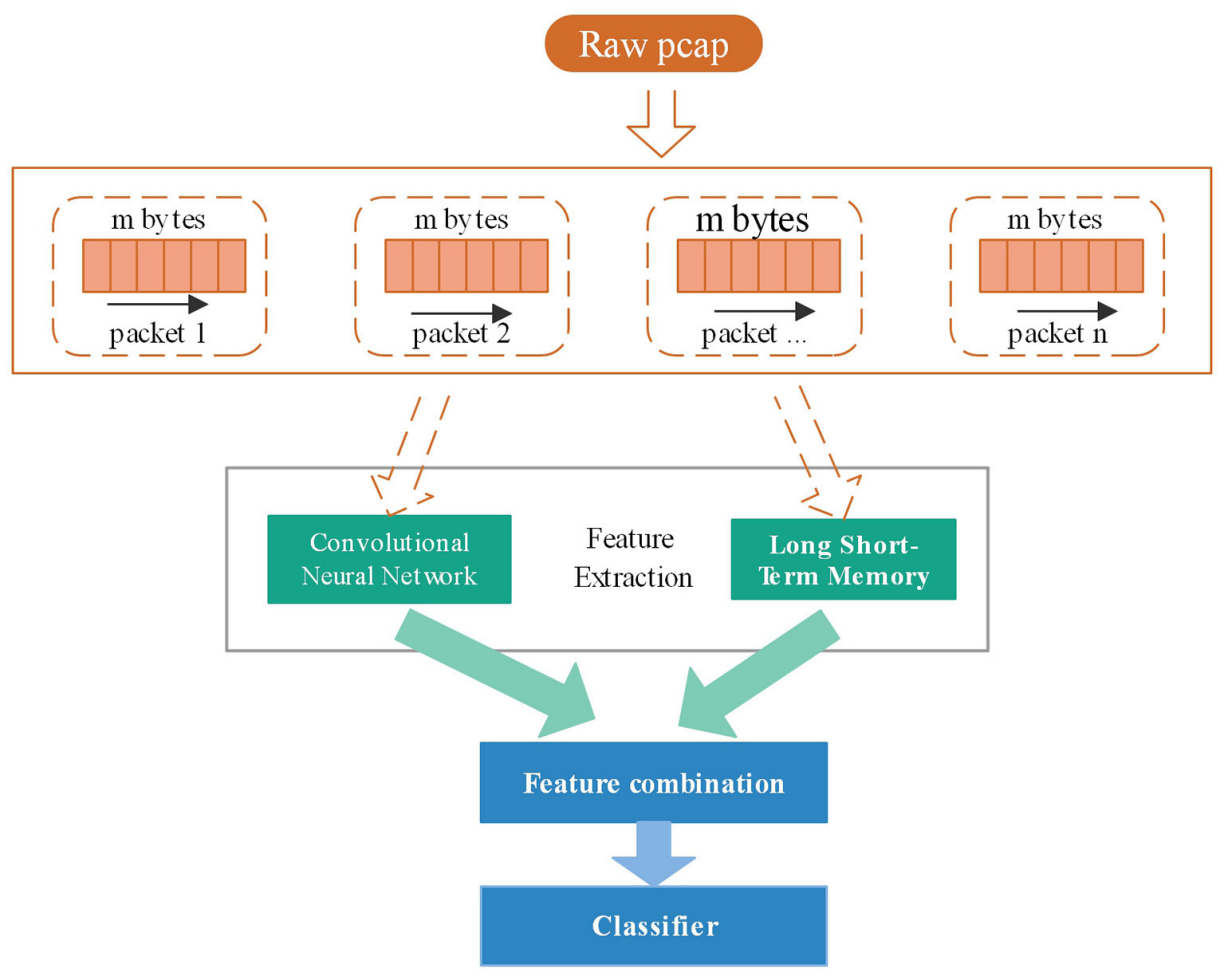
Convolutional neural network and LSTM neural network joint processing model.

A reasonable network structure plays an important role in the process of deep learning to identify encrypted traffic. This article draws on the idea of bagging, and designs a neural network with convolutional time series to identify encrypted traffic, as shown in Figure 4. The model has two inputs for the same sample. On the left side of the model, the input is the overall picture converted from the session bytes recombined from the data packet. This side of the model learns the structure information characteristics of the encryption suite of the traffic data; On the right side of the model, the input is bytes intercepted by multiple consecutive data packets in a session. The right side of the model learns the behavioral communication characteristics between traffic data, combines the two feature vectors together through splicing, and then passes through the neural network Layers are classified. Compared with the previous convolutional neural network and time series neural network, the combined neural network designed in this paper has an accuracy increase of nearly 4%, which further proves that a reasonable neural network structure is essential for the improvement of encrypted traffic recognition.

In Figure 4, two neural network structures are adopted to extract the different characteristics of encrypted traffic. The convolutional neural network on the left learns the overall structural characteristics of the traffic data packet, intercepting the first m data packets of the session, each The data packet intercepts n bytes, and each byte corresponds to two hexadecimal digital representations in the original traffic, which can be converted into a 1–255 decimal representation as input to obtain a matrix of size m^*^n. The process of the convolutional network refers to the network structure of session2, and uses multiple convolution kernels of different sizes to convolve the same data. The network structure is shown in Table 2.

**Table 2:** CNN network structure on the left.

**layer**	**Name**	**Activation function**	**Convolution kernel**	**Kernel number**	**Step**
1	Condv2	ReLU	3*3	256	1
2	Condv2	ReLU	3*3	128	1
3	Batch Norm	–	–	–	–
4	Inception-1	ReLU	1*1	128	1
4	Inception-2	ReLU	1*1	64	1
			3*3		
4	Inception-3	–	3*3	64	1
			1*1		
5	Inception-concact	–	–	–	–
6	PrimaryCaps	Squash	6*6*256	8	2
7	DigitCaps	Squash	–	–	–

The time series neural network used on the right is used to learn the communication timing characteristics between encrypted traffic data packets. Its input is the same as the data format on the left. It intercepts the first m data packets of the session, and each data packet intercepts n bytes. A byte corresponds to two hexadecimal digital representations in the original traffic, which can be converted into a 1–255 decimal representation as input, and a two-dimensional array is obtained, so that the input representation is the same as the natural language text analysis The input method of a sentence is the same. Compared with the traditional time series network structure, this paper adds a self-attention mechanism, so that it can capture the dependence of learning data packet communication behavior and learn more timing characteristics. The network structure is shown in Table 3. The output data of the network structure on the left and right sides are spliced together as the input of the classifier. This part of the network structure is shown in Table 4.

**Table 3:** LSTM network structure on the right.

**Layer**	**Name**	**Activation function**	**Weight matrix**
1	Elmo-1-Forward LSTM	ReLU	96*128
1	Elmo-1-ReverseLSTM	ReLU	96*128
2	Elmo-2-Forward LSTM	ReLU	128*128
2	Elmo-2-Reverse LSTM	ReLU	128*128
3	Elmo-concact	–	–
2	Two-Way LSTM	ReLU	128*256
3	Batch Norm	–	–
4	Self-Attention	–	–
5	Batch Norm	–	–

**Table 4:** Classification layer network structure.

**Layer**	**Name**	**Activation function**	**Length**
1	Concact	–	–
2	Full Connect	ReLU	512
3	Full Connect	ReLU	64
4	Full Connect	Softmax	7

## Result

The system of the experimental environment is Ubuntu 16.0, based on Keras running framework. RAM is 96G and video memory is 16G.

**Figure 5:**
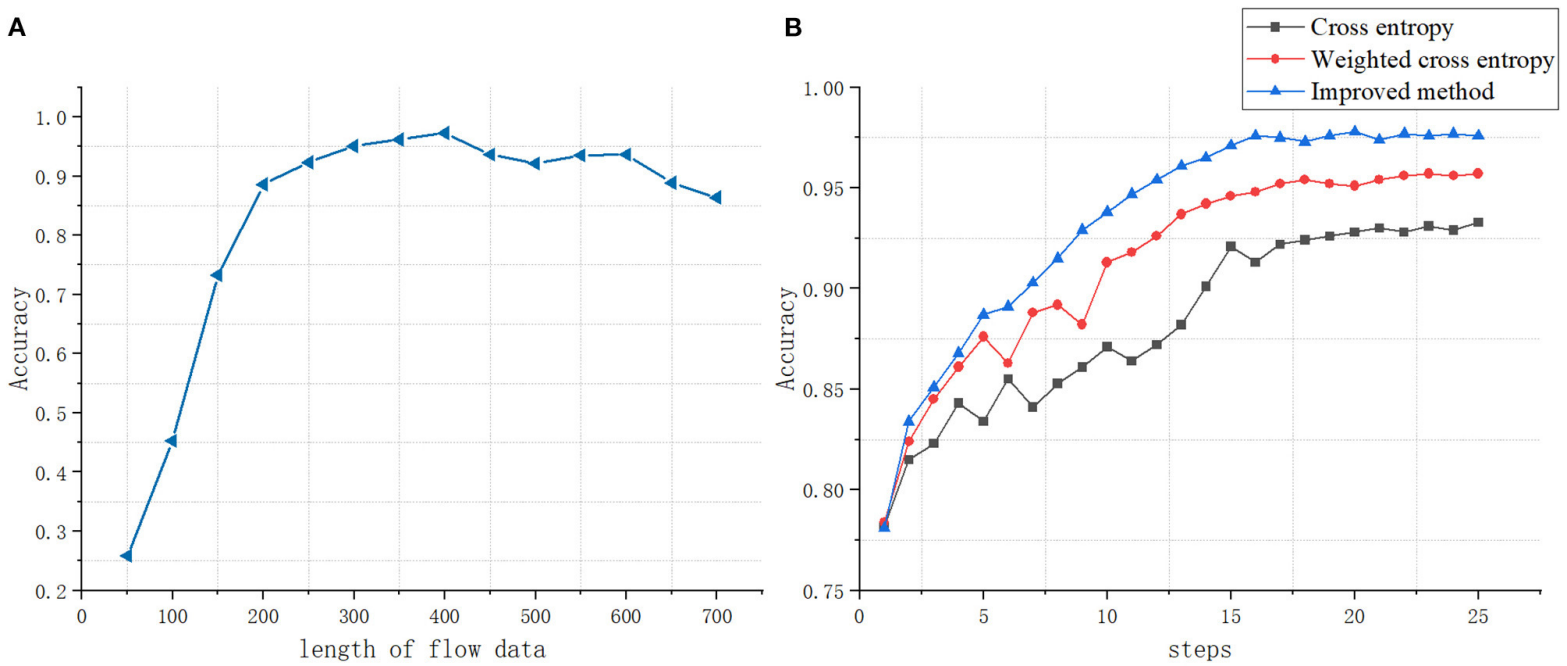
**(A)** The impact of data stream length on classification accuracy. **(B)** The impact of different loss functions on classification accuracy.

Different input byte length will affect the discrimination effect of the classifier, so choosing the appropriate byte input length is very important for the classifier. We have studied the impact of the byte input length from 50 to 750 on the classification results, as shown in Figure 5A. As the input byte length increases, the more features the classifier can use, the better the classification effect of the classifier. When the length increases at a certain threshold, the classification effect has not improved significantly. In order to save computing resources and time, we select the input data length as short as possible.

The choice of loss function will affect the classification effect of the classifier. The loss function calculates the distance between the probability distribution p and the probability distribution q predicted by the classifier. This article uses a loss function optimized based on focal loss *Loss(p,q)*=*L*.

$$\begin{array}{l} \text{L}=\overset{K}{\underset{i=1}{\sum }}\overset{K^{\prime }}{\underset{j=1}{\sum }}\alpha_{i}{\left(1-q\left(x_{ij}\right)\right)}^{\gamma }p\left(x_{ij}\right)\text{lg}\left(q\left(x_{ij}\right)\right) \end{array}$$

For a certain type of sample, the higher the value of *q*(*x*_*ij*_) is, the smaller the value of 1 − *q*(*x*_*ij*_) is, thus reducing the weight of this type of sample. The value of *q*(*x*_*ij*_) is small, and the 1 − *q*(*x*_*ij*_) will be large, which can increases the weight of the sample recognized hard. The value of α_*i*_ is inversely proportional to the number of samples of each type. Use the parameter γ to automatically adjust the ratio of the loss function. This not only considers the imbalance of sample categories in traffic identification but also solves the difference of recognization cost in different samples. Figure 5B shows the comparison with the cross-entropy loss function and the weighted cross-entropy loss function.

**Figure 6:**
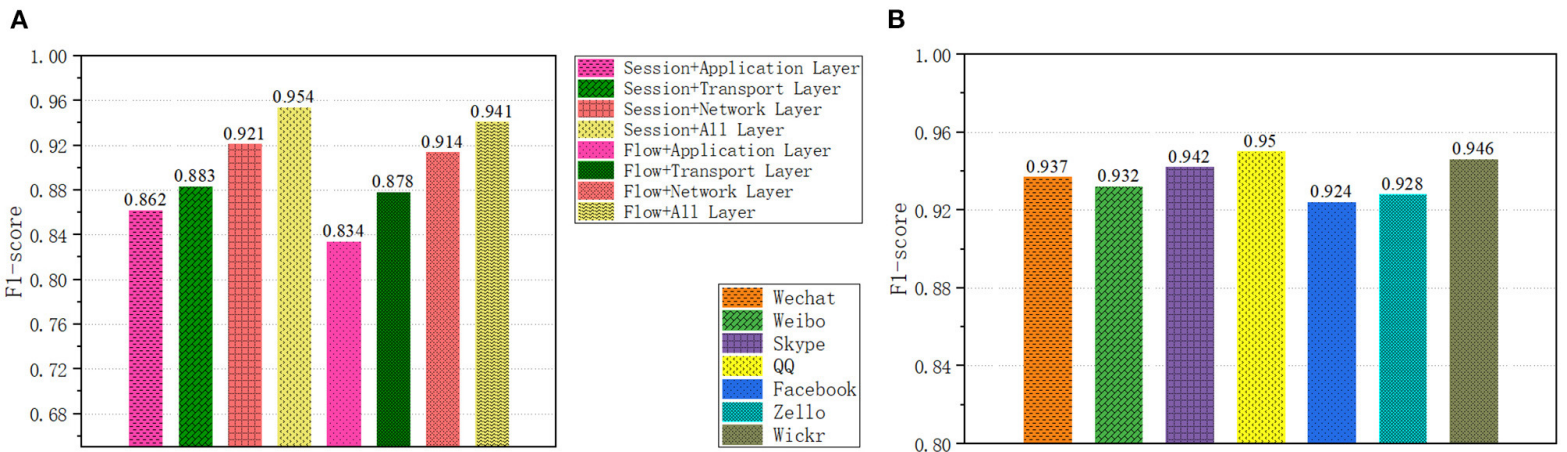
**(A)** Classification results of different network traffic forms. **(B)** Application classification results of convolutional neural networks.

Different traffic forms and network layer divisions contain different data content. We have studied the impact of different traffic forms and network layers on the classifier, as shown in Figure 6A. A total of eight types of samples were obtained for the two traffic forms and the four network layers. On the data set ISCX (Draper-Gil et al., 2016), we compared the recognition effects of the eight forms. As can be seen from Figure 6A, Session + All Layer performs best. The accuracy rate is 0.942, the recall rate is 0.973, and the F1-score is 0.955, because it contains more traffic characteristics.

**Figure 7:**
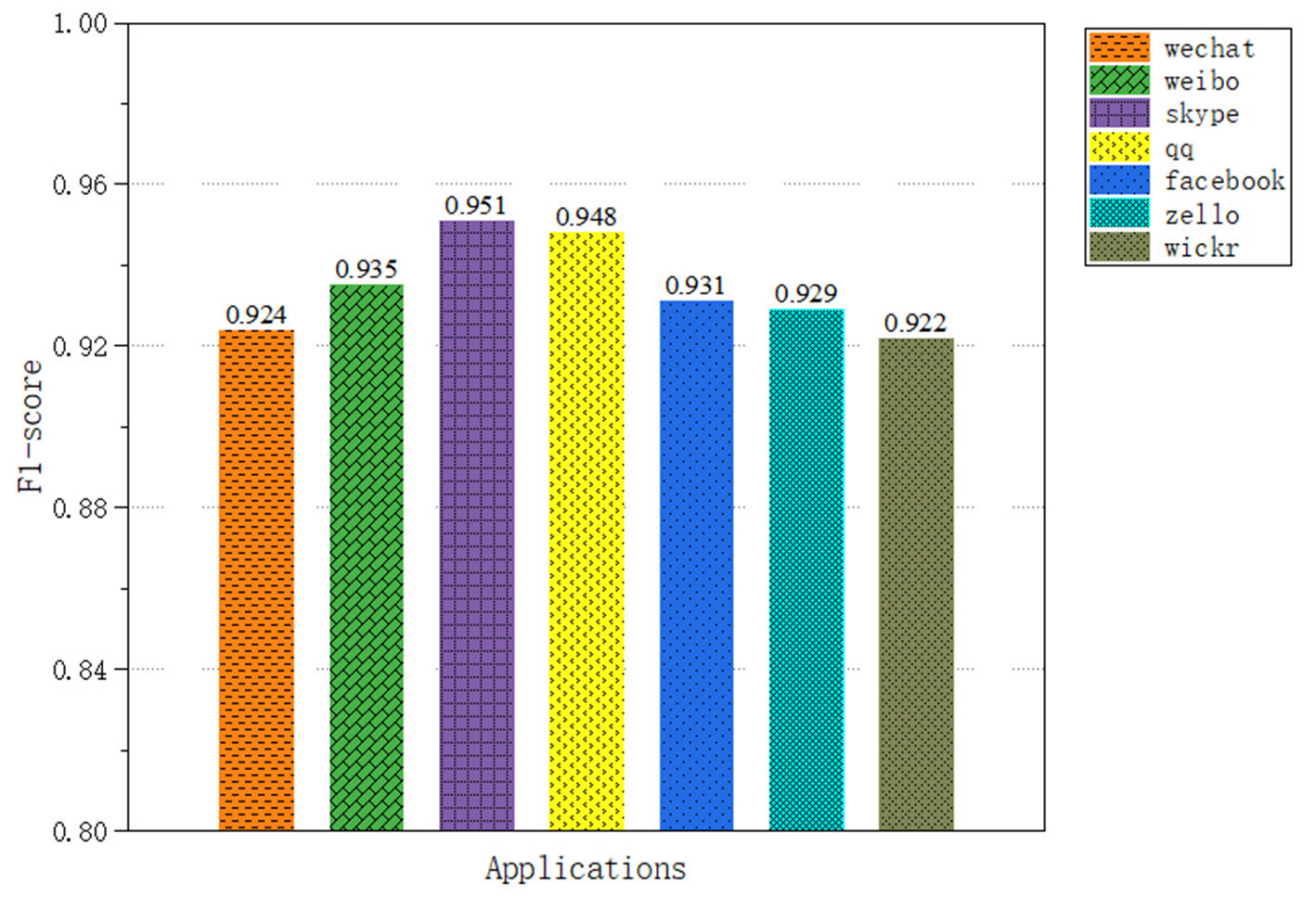
Application classification results of LSTM neural network.

In application classification, we selected seven applications, a total of 52,155 encrypted network traffic samples, with an average of 7,000 sample data for each application. The results of the capsule convolutional neural network are shown in Figure 6B and the results of Long short-term memory network are shown in Figure 7. The results show that these two methods have excellent performance for traffic classification.

Many scholars have performed classification method evaluation on the data set ISCX (Moore and Zuev, 2005; Alberto et al., 2006; Huang et al., 2014). Here we compare the Inception-CapsNet classifier with them. As shown in **Table 6**, it can be seen that the accuracy and recall rate of the other four types of methods have been improved. For the decision tree C4.5 algorithm, the accuracy rate has increased by 4.3%, and the recall rate has increased by 7.0%.

**Figure 8:**
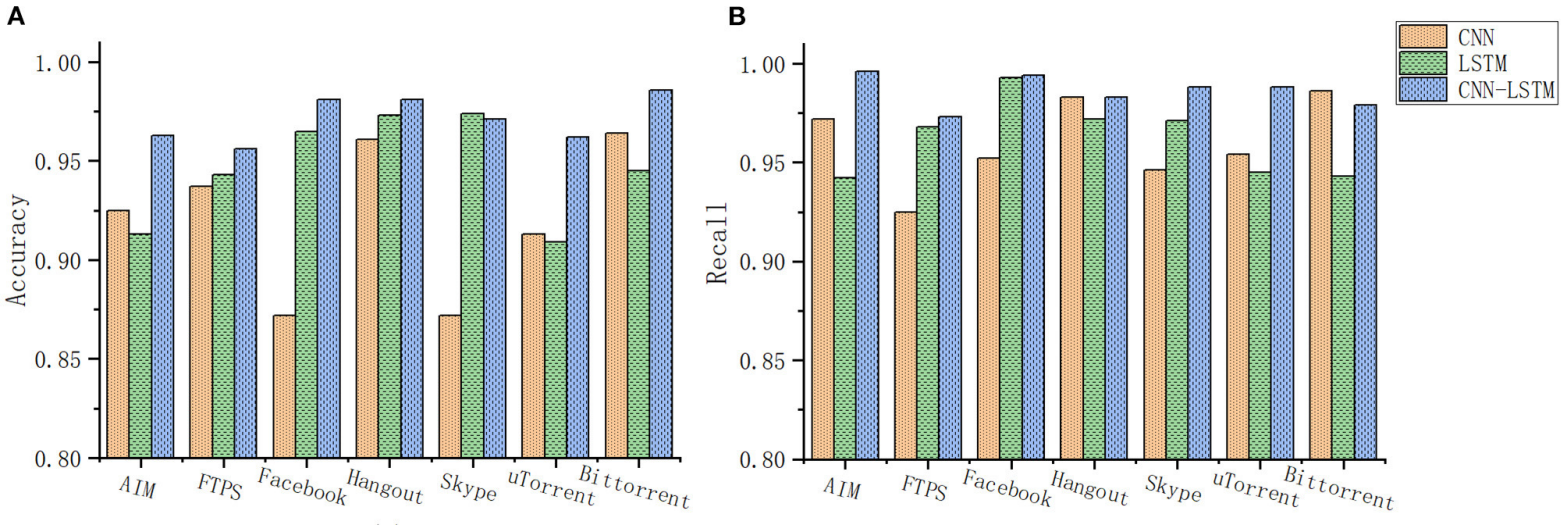
**(A)** CNN-LSTM joint network application classification accuracy rate. **(B)** CNN-LSTM joint network application classification recall rate.

The CNN-LSTM joint model experimental results are shown in Figure 8. For Hangout and Bittorrent, the convolution method has a high recognition accuracy of 96%. For Facebook, Skype has a low recognition accuracy rate of only 87%, while for ATM, Hangout, and Bittorrent, The recall rate is high, reaching 98.

The results show that the recognition accuracy of the time series method for Facebook, Skype, Hangout is as high as 97%, and the recognition accuracy for AIM and uTorrent is low, only 91%. Facebook's recognition recall rate is even higher, reaching 99%.

For the combined model, the recognition accuracy has been significantly improved. Among them, Facebook, Hangout, and Bittorrent have a high recognition accuracy of 98.5%; for ATM, FTPS, Skype, and uTorrent, the recognition accuracy is high. Reached 96%; and for the recall rate, the recall rate of encrypted traffic of AIM, Facebook, Hangout, Bittorrent, and Skype reached 99%. For the traffic FTPS, the recall rate of the two encrypted traffic of uTorrent reached 96%.

From the above results, it can be seen that different network structures extract features of different dimensions, and have different preferences for the quasi-curvature and recall rate of encrypted traffic recognition of the same application. For some traffic convolutional neural networks, the recognition accuracy rate is higher. But the recall rate may be relatively low, such as FTPS. For the encrypted traffic of this kind of application, although the recognition quasi-curvature of the time series neural network is moderate, the recall rate is high. In the experimental results of, this conclusion has also been proved, the accuracy rate of its encrypted traffic has reached more than 96%, the recall rate is 99%, only two kinds are lower, and it is also more than 96%. The effect is compared with the previous neural network. The organization has very distinctive features. This proves that extracting features from different dimensions and different feature spaces helps to capture more recognizable features and enhance the model effect.

Table 5 shows the comparison results on ISCX, where the number of features used by the machine learning method is different, reflecting the complexity of the manual design, such as SVM using 21 manual design Features. The decision tree uses 18 artificially designed features, but the two methods have only ten common features, and it is difficult to generalize to other data sets. Table 6 shows the comparison results on the UNIBS data (Gringoli et al., 2009). In this data set, the results obtained by the relevant literature are given by the SVM model, but the accuracy of the combined model exceeds the model, and the recall rate is far higher. For SVM, it is 0.05 less than the random forest model with the best recall rate, ranking second, and the overall effect is very good. The results show that the accuracy of the CNN-LSTM network is 8% higher than other methods.

**Table 5:** Comparison of classification performance with other traffic classification algorithms on Datasets ISCX.

**Algorithm**	**Accuracy**	**Recall**
CNN-LSTM	0.981	0.995
C4.5	0.901	0.903
SVM	0.943	0.929
1dCNN	0.933	0.951
2dCNN	0.936	0.955
Apriori	0.931	0.911
Naïve Bayes	0.911	0.927
Hmm-crf	0.955	0.967

**Table 6:** Comparison of classification performance with other traffic classification algorithms on datasets UNIBS.

**Algorithm**	**Accuracy**	**Recall**
LSTM	0.945	0.973
SVM	0.959	0.953
Multi-classifier	0.924	0.971
Random forest	0.936	0.992
Xgboost	0.931	0.961
CNN-LSTM	0.986	0.987

## Conclusion

In this literature, we propose a capsule convolutional neural network joint the long short-term memory network traffic classification framework. For the problem of imbalance of sample categories in flow recognition, an objective function related to weight and sample recognition accuracy is designed to reduce the classification impact caused by sample imbalance. Besides, the inception structure is added to allow the model to learn diverse features, and the capsulenet structure is added to allow the model to learn the correlation of high and low dimensional features. This model can automatically identify a variety of encrypted traffic and seek the global optimal classification result. The experimental results show that this method can effectively classify the encrypted traffic and is better than previous research work. At the same time, our work proves that the optimized neural network structure can achieve better recognition results.

As future work, We believe that we should try to pay attention to the characteristics of network traffic of different behaviors, so as to more comprehensively describe the communication process between users and robots.

## Data Availability Statement

The original contributions presented in the study are included in the article/supplementary material, further inquiries can be directed to the corresponding author/s.

## Author Contributions

MG contributed the idea of the paper, verified the idea through experiments, and wrote the paper at the same time. XY guided the idea of the paper and LL assisted the experiment. All authors contributed to the article and approved the submitted version.

## Conflict of Interest

The authors declare that the research was conducted in the absence of any commercial or financial relationships that could be construed as a potential conflict of interest.
